# Phenotypic Presentation of Children with Joint Hypermobility: Preclinical Signs

**DOI:** 10.3390/children12010109

**Published:** 2025-01-18

**Authors:** Mateus Marino Lamari, Neuseli Marino Lamari, Michael Peres de Medeiros, Gerardo Maria de Araújo Filho, Adriana Barbosa Santos, Matheus Gomes Giacomini, Vitor Roberto Pugliesi Marques, Eny Maria Goloni-Bertollo, Érika Cristina Pavarino

**Affiliations:** 1 Department of Epidemiology and Public Health, Medical School of São José do Rio Preto (FAMERP), Av. Brigadeiro Faria Lima, 5416, Vila São Pedro, São José do Rio Preto 15090-000, SP, Brazil; mateus.lamari@edu.famerp.br; 2 Department of Neurological Sciences, Psychiatry and Medical Psychology, Medical School of São José do Rio Preto (FAMERP), Av. Brigadeiro Faria Lima, 5416, Vila São Pedro, São José do Rio Preto 15090-000, SP, Brazil; michael.medeiros@edu.famerp.br (M.P.d.M.); gerardo.filho@famerp.br (G.M.d.A.F.); 3 Department of Computer Science and Statistics, São Paulo State University (UNESP), R. Cristóvão Colombo, 2265, Jardim Nazareth, São José do Rio Preto 15054-000, SP, Brazil; adriana.barbosa@unesp.br; 4 Foundation of the Regional Medical School of São José do Rio Preto (FUNFARME), Av. Brigadeiro Faria Lima, 5544, Vila São Pedro, São José do Rio Preto 15090-000, SP, Brazil; matheus.giacomini@edu.famerp.br; 5 Epilepsy Surgery Center (CECEP) of the Hospital de Base de São José do Rio Preto, São José do Rio Preto 15090-000, SP, Brazil; neurologia@santacasasaocarlos.com.br; 6 Genetics and Molecular Biology Research Unit, Department of Molecular Biology, Medical School of São José do Rio Preto (FAMERP), Av. Brigadeiro Faria Lima, 5416, Vila São Pedro, São José do Rio Preto 15090-000, SP, Brazil; eny.goloni@famerp.br (E.M.G.-B.); erika@famerp.br (É.C.P.)

**Keywords:** joint instability, range of motion, joint mobility, hypermobility, child, pediatrics

## Abstract

Introduction: Joint hypermobility (JH) is mobility beyond the normal range of motion. JH can be an isolated finding or a characteristic of a syndrome. Characteristics related to the sitting position with atypical body positions, such as sitting in splits (S), with the foot on the head (F), in W (W), in a concave shape (C), episodes of dislocations, and subluxations, suggest impacts on body mechanics since childhood, with damage to the conformation of the joints. Objectives: Identify preclinical signs of JH, in addition to Beighton Score (BS), through signs that are easily recognized early by pediatricians and family members to avoid possible joint deformities in the future. Methods: The medical records of 124 children (59.7% girls) between one and nine years old were analyzed. JH was assessed using the BS, a history of luxations/subluxations, and the concave (C), “W”, “splits” (S), and foot (F) on head sitting positions. Results: The concave sitting position was the most common, followed by W, F, and S in decreasing order. A total of 52.4% of the children had BS > 6, with a higher prevalence among girls (60.8%) compared to boys (40.0%); a difference statistically significant (*p* = 0.024, Fisher’s exact test). Thirty-two patients (27.4%) had luxations/subluxations with the higher scores. Conclusions: Sitting in S, F, W, and C positions are preclinical phenotypic characteristics of JH, easily identified by pediatricians and family members to prevent possible joint deformities. BS ≥ 6 is more frequently observed in all positions. The majority of the total sample has BS > 6, with a significant female gender influence. Among those with a history of occasional joint dislocations and subluxations, half of them have the highest BS scores.

## 1. Introduction

Joint hypermobility (JH) refers to the ability of the joint to move beyond normal limits along physiological axes [1]. It is also considered a deviation from normality, genetically determined, with influences from age, sex, and ethnicity [2].

The frequency in the general population varies between 2% and 64.6% across different groups [3,4,5]. It may be present without complications, especially in children [3,6,7], and can have potential functional impacts both at home and at school [8]. Therefore, JH is a descriptor and not a diagnosis, and in recent years, increasing attention has been given to JH and related secondary disorders, since it predisposes to an excess of macrotrauma and microtrauma [9]. JH I identified using the Beighton Score [10], which is considered the most reliable and valid tool to identify generalized joint hypermobility (GJH) [11], which is defined by the presence of JH in the limbs and axial skeleton concomitantly [9], which is scored from zero to nine, with BS ≥ 6 being a marker of GJH in children. However, recent studies suggest that adjustments should be made to consider JH’s condition in a generalized way [5]. JH may present among the characteristics of major, rarely diagnosed syndromic conditions [12], such as Ehlers–Danlos Syndromes (EDS) and Hypermobility Spectrum Disorders (HSD) [9]. The phenotypic boundaries of JH are still not well-defined [13].

Several factors influence joint mechanics, including genetic, biological, hormonal, nutritional, and common stress factors, which contribute to adaptation to present forces [14]. Additionally, other external factors can affect the phenotype [14,15]. Thus, the evolution of body movements occurs in response to the adaptive mechanical forces of the musculoskeletal system’s tissues in joint mobility [16], while also considering environmental situations [17] partly responsible for the architectural structure of the body in response to force [18].

There are several stages of growth characterized by the evolutionary process of the human species from birth to adulthood [19], starting from conception and continuing through the first two decades of life [20]. This is a dynamic process resulting from the increase in cell number and size, leading to progressive changes in height and weight, and occurs while the cartilages of the long bones have not yet ossified and closed [15,21].

In this context, postural control is impaired in individuals with JH due to excessive joint movement, with loose ligaments unable to align the body properly [22,23,24]. This condition results in abnormal stress on the joints [25], leading to inadequate postural habits [26,27], pain [28], microtrauma [29], and macrotrauma due to occasional subluxations and dislocations [30]. The asymmetric loading of joint surfaces contributes to the premature wear of the articular surface [30].

A recent study involving children, adolescents, and adults [31] investigated motor characteristics related to JH, such as the current habit of sitting in a “concave” position, which totaled 54.15% of them. They accounted for the vast majority (69.92%) when also considering those who were able to sit concavely in the past. The study also found that 39.21% always sat in the “W”. These also accounted for the majority (55.81%) when including those who were previously able to sit in a “W” position.

As inadequate postural habits become more pronounced with the force of gravity and are associated with the natural process of tissue growth and maturation, they undergo structural changes until growth in height is complete [32]. Physical abnormalities are observed through deformities in the locomotor system in children, youth, and adults with JH, with severe consequences for the elderly [33]. Many individuals have undergone avoidable surgical interventions, which could have been identified and addressed with proper healthcare during childhood and/or adolescence [7,34].

There are several intervening factors in joint mechanics that can influence the phenotype [14,15] through responses to adaptive mechanical forces [16], and therefore have an impact on the body’s architectural framework [18]. One is joint hypermobility (JH), a condition that affects postural control [22,23,24] due to inadequate postural habits [26,27] and joint instability resulting from microtraumas [29] and macrotraumas [30] during tissue growth and maturation [32]. Early detection of JH during childhood or adolescence is essential [7,34]. Early identification of pre-clinical signs, which may progress to deformities together with early treatment, is justified [33].

In this context, we seek to identify preclinical signs of JH in addition to BS through signs that can be easily recognized early by pediatricians and family members to avoid possible joint deformities in the future; analyze the distribution of BS in the total sample and according to sex; verify the association between BS ≤ 6 and BS > 6 and the categories in the total sample and according to sex; investigate the ability to sit in these positions in the present and in the past. Additionally, we estimate the percentage distribution of the ability to sit in the S, F, W, and C positions, according to BS in the total sample as to sex; and evaluate the occurrence of joint subluxations and dislocations in the total sample and their association with BS.

## 2. Methods

This was a cross-sectional, observational, quantitative, retrospective study, carried out in São José do Rio Preto, SP, Brazil, using clinical records about JH from the Lamari Clinic, Ltd. In this study, patient records with information of interest for the data-collection instrument were the inclusion criterion. The ethical approval was obtained from the Human Research Ethics Committee of the Medicine School of São José do Rio Preto (FAMERP), with approval certificate number 36145820.6.000.5415, in accordance with regulatory norms established in Resolution 466/2012 of the Brazilian National Board of Health.

### 2.1. Data Collection Instrument

For the study, the researchers determined an instrument to record the data, which were collected from the records of hypermobile patients referring to the period from January 2014 to March 2020, considering the results of general and specific physical examinations as well as characteristics associated with JH, a family history of JH, and the BS [10].

### 2.2. Examination of Sitting Positions

**Figure 1:**
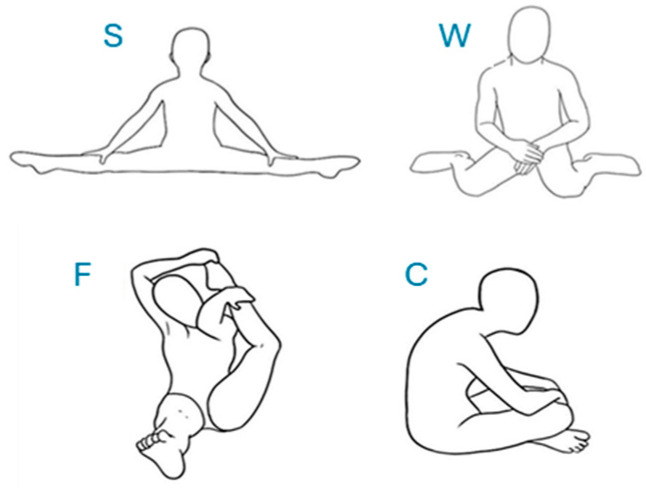
Illustration of four sitting positions with atypical body positions, such as sitting in splits (S), with the foot on the head (F), in W (W), in a concave shape (C), for assessment of JH of hips and trunk. Original image from the doctoral thesis of the first author.

JH was assessed in the sitting position, with the analysis of the following abilities (observed or reported in the present or past), illustrated in Figure 1:

S: (“splits”) limbs abducted at 180°;

W: limbs abducted with knees at complete flexion and feet turned outward;

F: foot on head;

C: (concave) forward lean of trunk and head with shoulder and abdomen protruded.

The characterization of JH in the four seated positions C, W, E, and P is evaluated and scored individually. One point is awarded for each positive result that defines the individual as capable of maintaining the posture, and zero points are given when they are unable to do so. The total score from the four positions can range from zero to four. The ability to execute one or more of the four positions indicates the need for referral to physiotherapy for inhibition and re-education of the identified posture(s).

### 2.3. Examination of the GJH Utilizing BS

Five different body locations were examined in order to determine JH based on BS. For every favorable outcome on either side, one point was awarded. The BS, which varied from 0 to 9, was calculated by adding all of the points, with BS ≥ 6 being a marker of GJH in children and prepubescent adolescents. The following aspects were investigated: passive extension of the fifth finger (F), passive apposition of the thumb on the forearm (A), active hyperextension of the elbow (E) and knee (K), and anterior trunk flexion (ATF), except for the spinal column variable. The data were recorded using the data collection instrument. The measurements of the articular amplitude of the variables F, E, and K were obtained by the manual goniometer [35].

The following configurations are considered to score the GJH: 1. 5th finger hyperextension is defined by passive extension with an angle > 90°; 2. wrist hyperflexion is defined by passive thumb touch to the forearm flexor region; 3. elbow hyperextension is defined by active extension with an angle > 10°; 4. knee hyperextension is defined by active extension with an angle > 10°; 5. anterior spinal column hyperflexion is defined by palms touching the ground with extended lower limbs.

### 2.4. Identification of Occasional Occurrence of Joint Dislocations and Subluxations

Patients and/or family members received guidance to understand the possible occurrence of episodes of dislocation, which is considered the complete joint displacement from the normal position of one or more joints, with the joint surfaces losing contact with one another. While articular subluxation is considered the occurrence of misalignment or partial displacement of one or more joints without the joint surfaces losing contact with one another [36]. Data were obtained through self-reports. The confirmation of one or more occasional joint dislocations or subluxations in a child with JH, regardless of the body region, indicates the need for physiotherapy assistance for joint stabilization.

### 2.5. Statistical Analysis

Analyzing data required the calculation of frequencies and descriptive statistics to provide a global view of the sample analyzed from different perspectives. Comparisons were conducted using non-parametric tests. The Mann–Whitney test was used to compare continuous variables between groups since it is appropriate for independent samples that do not have a normal distribution. For proportion comparisons between the sexes, Fisher’s exact test was utilized in accordance with the Beighton Score categories. Additionally, for analyses involving multiple observations per individual, such as those related to different sitting positions, statistical tests that assume independent observations, like Chi-square or Fisher’s exact test, are not recommended. All analyses were conducted with a significance level set at 5% (*p* < 0.05). Basic graphs complement the numerical results [37]. Analyses were performed in Minitab v.16 [38].

## 3. Results

The sample was composed of 124 children one to nine years of age [mean age: 5.5 ± 2.4 years; 74 girls (59.7%) and 50 (40.3%) boys] with the majority of the participants from the state of São Paulo, accounting for 84.7%. The state of Minas Gerais represented 4.0%, Mato Grosso 1.6%, Rio de Janeiro 2.4%, Rondônia 2.4%, and other states 4.8%. In the total sample, 59.7% were women, with the mean and median age of the girls being seven years and a mode of 8 ± 1.81 years. For the boys, the mean age was six years, with a median of seven years and a mode of 8 ± 2.24 years.

### 3.1. Postures and Abilities in Sitting Position

**Figure 2:**
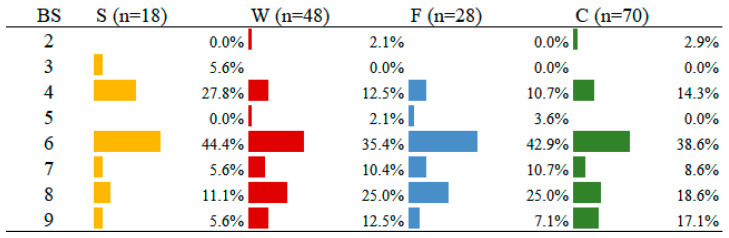
Percentage distribution of BS per each sitting position sitting in splits (S), with the foot on the head (F), in W (W), in a concave shape (C) for assessment of JH of hips and trunk. Original image from the doctoral thesis of the first author.

The concave (C) sitting position was the most frequent in the sample (*n* = 70), followed in decreasing order by W (*n* = 48), F (*n* = 28), and S (*n* = 18). Figure 2 shows the percentage distribution of BS per position S, W, F, and C. In general, there was a similar percentage distribution for the different positions, with a considerable part of the children with BS ≤ 6 for all sitting positions.

**Figure 3:**
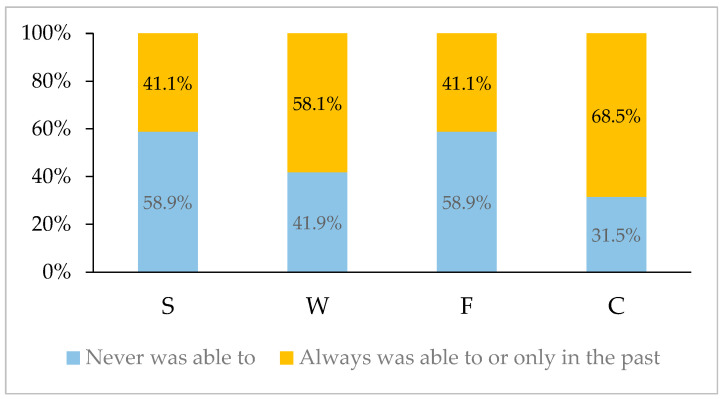
Percentage distribution of the total sample for those who were never able to sit in splits (S), with the foot on the head (F), in W (W), in concave shape (C) positions, and those who were able to do so either currently or in the past.

Of the 124 patients, 6 (4.8%) were able to sit in all four positions. Figure 3 illustrates the percentage distribution of patients’ ability to perform the sitting positions, considering both past and present abilities. The results indicate that most children (58.9%) were never able to sit in the S and F positions. In contrast, the W (58.1%) and C (68.5%) positions were most frequently performed, with most children either consistently able to perform these positions or able to do so at least in the past. Additionally, when analyzing positions that could only be performed in the past, S was the most frequent, followed by W, F, and C.

### 3.2. Influence of Sex on BS

Descriptive statistics related to BS were calculated to provide a global view of the sample analyzed (Table 1). The results of medians reveal that 50% of girls presented BS from 7.0 to 9.0, in comparison with boys the range was 6.0 to 9.0. The dispersion of BS values was similar between sexes in terms of range, standard deviation (s), and coefficient of variation (CV). The CV was calculated to express the proportional variability of BS relative to the mean ($\bar{x})$. The Mann–Whitney U test also exhibited in Table 1 revealed a statistically significant difference between sexes (*p* = 0.046), suggesting that hypermobility was more prevalent among females.

The BS ranges were classified into two ranges: BS ≤ 6 and BS > 6, based on the established literature, which identifies a BS > 6 as a relevant marker of generalized hypermobility in children. This threshold valuer is aligned with existing studies and facilitates the comparison of hypermobility prevalence between sexes.

The results presented in Table 2 highlight that a total of 65 children (52.4%) had BS > 6. Furthermore, the data provide evidence of an association between sex and BS range. For girls, 45 (60.8%) had a BS > 6, while for boys, this was 20 (40%). This difference found between sexes was statistically significant according to Fisher’s test (*p* = 0.024).

Additional descriptive results presented in Table 3 are related to the sample stratification by sex in reference to children who have always been able to sit in S, W, F, or C positions. For all positions, female children present a higher prevalence, reinforcing this as a characteristic of generalized hypermobility in children.

As a complementary result, the analysis of the total sample revealed that 5.6% of the children had luxations while 21.8% had subluxations. Notably, 50% of these children had the highest BS (8 and 9).

## 4. Discussion

JH is a risk factor for musculoskeletal issues in pediatrics [31,33], which can be attributed to impaired postural control due to excessive joint movement, loose ligaments, and an inability to properly align the body [26,27]. This condition leads to abnormal stress on the joints [25], resulting in inadequate postural habits [26,27], pain [28], and potential deformities [30,31,32,33]. These characteristics are often noted by parents from an early age but may be overlooked by healthcare professionals as preclinical signs of JH, as observed in the sample of the present study.

Lack of awareness about the impacts of JH also extends to the educational setting, where children may remain seated for extended periods on school benches, experiencing pain and fatigue [39]. A recent literature review [40] supports the notion that children with JH may exhibit high levels of motor and coordination difficulties, with functional impacts at home and at school [8]. Consequently, children with developmental coordination disorder may present JH as a contributing factor to these difficulties [8,41]. The atypical sitting positions identified in this study could serve as an easily observable reference for JH, even by teachers.

During adolescence, the musculoskeletal system structures finalize for adulthood, which underscores the importance of observing even minimal preclinical signs of deformities in children. The outcome of health care will be influenced by the time of evolution and the start of interventions [33].

Characteristics related to JH, such as sitting in C, F, S, and W positions, are easily noticeable in children, as shown by the present study. These could lead to consequences for the locomotor system, including microtraumas [29] and macrotraumas [30], progressing to deformities, chronic pain, reduced mobility, and physical disability [30,31]. Musculoskeletal conditions have a high potential for causing disabilities [33] due to compensatory movements, which increase the risk of muscle, ligament, and capsule injuries by impairing their physiological properties from an early age [31,33]. In this study, the C sitting position exemplifies this condition.

Hypermobile joints are susceptible to dislocations and subluxations along non-physiological axes, exacerbated by joint instability (JI) [31]. These issues are often worsened by microtraumas—subtle and typically unnoticed injuries—as well as occasional dislocations in both large and small joints [30]. This asymmetrical load on the articular surfaces contributes to premature wear. Over time, this can predispose individuals to recurrent pain, often beginning in childhood as “growing pains” localized in the lower limbs, a phenomenon reported by more than one-third of the total sample in a study conducted in Brazil with children, adolescents, and adults [31]. In this context, “growing pains” may be indicative of pain associated with JH in children. This condition requires population studies with control groups. Microtrauma in hypermobile individuals may persist and evolve into early joint degeneration, known as early-onset osteoarthritis [9]. However, early identification of these characteristics and physiotherapeutic intervention in childhood can prevent these potential complications [6,7,24]. This study revealed that between 5.6% and 21.8% of the sample had dislocations/subluxations, and 50% of these patients had the highest scores on the BS, between 8 and 9. Therefore, these patients are more susceptible to repeated microtraumas and macrotraumas, as well as to manifestations of pain and deformities.

Early recognition and preventive actions can significantly alter the course of this condition, especially since musculoskeletal manifestations are intensified until the end of height growth [32,33]. Clinical practice and our studies show that parents often express concern about their children’s unusual postures and contortions, seeking help that is frequently downplayed by healthcare professionals. Recent studies also indicate a connection between JH and autism spectrum disorders (ASD), as well as attention-deficit/hyperactivity disorder (ADHD) [42,43]. This reinforces the need for early identification of signs in childhood to understand these manifestations also in patients with ASD [43].

Additionally, it is important to consider that physical abnormalities emerge as inadequate postural habits are repeated, influenced by the force of gravity and the natural process of tissue growth and maturation, with potential structural manifestations until the end of height growth [32].

This study observed that inadequate postural habits, such as sitting in atypical positions and using unconventional body skills, are related to the hips and spinal joints. These conditions are common and indicate that postural control is impaired even in childhood. As a result, some of these children may develop a series of musculoskeletal complications, as bodily signs often intensify rapidly during adolescence and persist into adulthood. Knowing the diagnosis and interventions are important conditions for reducing morbidity and the costs of social and health services [33].

Age and gender may influence flexibility. Anatomically, according to Alter [44] and Malina and Bouchard [20], women generally have greater flexibility across all ages and pelvic differences compared to men. This study identified that women have a higher prevalence of JH characteristics according to the BS, as well as atypical postures/body skills investigated.

In the study by Lamari et al. [3], 1120 children between 4 and 7 years old of both sexes were assessed using the BS, which showed that even within age groups with minimal age variation, joint mobility decreases with increasing age. A similar result was observed in the present study regarding the ability to sit in S, F, W, and C positions, which were only performed in the past. The results indicated that these are characteristics identified in hypermobile children of both sexes, including those observed only in the past. This finding is suggestive for further studies. Although it is well documented that GH decreased with increasing age, it is still unclear which children remained with the GJH trait, and which will experience stiffness post-puberty.

The wide variation in the prevalence of the JH characteristic up to 64.6% with different populations implied greater difficulties in understanding the prevalence of this characteristic, with the exception of children and females, for whom there is ample research [3,6,31,34]. Our study included girls and boys with JH and the majority were women. Castori et al. [45] report that the higher frequency of females is associated with muscle mass composition and ligament stiffness, which provides greater joint stability in males. They also associate early health-seeking behavior with females. The results of this study corroborate the specialized literature by indicating that the prevalence of JH occurs in females even within a narrow age range. Analysis of the variables and the total BS score shows that sex influenced only the variable T.

GJH is defined by the BS [10], which is considered the most reliable and valid observational tool for both pediatric and adult populations [11], and remains the most commonly used by specialized literature [5,7,46], including over the past 50 years [33]. Using this tool, the analysis of GJH in our study showed that a BS ≥ 6 predominated among children of both sexes, with a localized predominance only in the upper limbs. Other studies have shown similar results [5,31]. There is a need for studies with similar populations, once GJH is among the diagnostic criteria for hEDS [2], with a score of BS ≥ 6 being a reference for prepubescent children and adolescents.

The characteristics of JH are little known, and even less so are its clinical implications, which use the BS, which is the tool commonly used in daily clinical practice, as well as research for its identification [11]. However, it is a screening tool for epidemiological studies and not for clinical use [47]. The analysis of sitting posture and unconventional exuberant presentations in children with JH are not analyzed, despite involving the hips and spine. Thus, there are no population studies to discuss our results. The exception is the study of a sample in different age groups [31].

Hypermobile children up to six years of age may exhibit musculoskeletal characteristics resulting from JH, which are subtle and perceptible and become more evident with growth and tissue maturation [3,4,31,48]. In pediatric clinical practice, JH-related conditions can be easily identified, many of which are preventable if identified early. In this study, hypermobile children predominantly showed high BS scores, as well as atypical body contortions and sitting postures with extreme, dysfunctional, and unviable ranges for the locomotor system mechanics. However, recognizing JH in routine pediatric practice can lead to early detection and actions to promote and prevent deformities, predominantly in the spinal mechanics, hips, and lower limbs [33].

It should be considered that the evolution of body movements occurs through the mechanical adaptive responses of the musculoskeletal system tissues, in form and function, to make them better suited to the environment [17]. It is important to emphasize that throughout human development, bones are subject to adaptations to loads and forces imposed on them. Thus, the combination of these mechanical properties and musculoskeletal tissue behaviors is relevant for understanding joint mobility functions [16]. It is emphasized that biomechanics shows that all human movement is the result of the forces around a joint [14] and that the body’s architectural framework is a physiological response to force [18]. Considering these biomechanical implications, the present study identified dysfunctional sitting positions that require early physiotherapeutic intervention to inhibit these inappropriate patterns from early childhood.

A recent study [31] with different age groups of patients with JH, of both sexes, found that the ability to sit in the C position currently and/or in the past was possible for most (69.92%), with no significant difference in the total sample (*p*-value = 0.556) or by age group (*p*-value = 0.054). In our study, the results of the analysis for the C sitting position were also present in the majority (68.5%), considering those who always exhibited this characteristic and those who exhibited it only in the past. In the same 2022 study [31], the authors identified that, when considering those who were always able or were able only in the past to sit in the W, in the majority (55.81%) of the total sample, the statistical difference was not significant, but it showed a significant difference when the analysis was carried out by age group. Analysis of the total sample in the present study also shows that the majority of children sit or have sat in the W (58.1%). The results of both studies were similar despite different age groups. No other studies were found in the specialized literature that corroborated these findings. Given the prevalence of these postures among individuals with JH, it is suggested to include these four sitting position variables in GJH assessments, as well as population studies with these inclusions.

Considering the influence of joint mechanics on the condition of individuals with JH in the sitting position, muscle weakness could justify the impairment in trunk stabilization, hip stability, and postural correction, which instinctively leads individuals with JH to sit in the W position. This position may cause the femur and tibia to undergo internal rotation and femoral anteversion, resulting in an increased base of support and trunk stabilization, which provides a sense of comfort. This comfort could also explain the high prevalence of the W position variable in this study.

The different factors influencing joint mechanics justify the concern with adaptive postures observed in hypermobile children, as shown by the analysis of the C sitting posture, which is most frequent among children with JH in this study. This posture suggests that vertebral stability requires static elements for the dynamic maintenance of each vertebral body on top of each other. It is also noted that JI is a primary cause of vertebral pathologies such as disc degeneration, osteoarthritis, spondylolisthesis, among others, and consequently pain [30].

In hypermobile individuals, ligamentous failure is characterized by inadequate tension to promote stability, and muscular deficits prevent adequate static and dynamic stability of the vertebral bodies. Thus, the weight of the trunk influences its anterior inclination due to the lack of antigravity muscles to keep the trunk upright and the deficit in muscles that provide pelvic vertebral segmental stability. Therefore, when considering the most relevant muscles of the lumbar spine, weakness of the erector spinae, transverse spine, latissimus dorsi, and quadratus lumborum can also justify failure to support the trunk [49], leading to undesirable postures in an attempt to realign and support body posture.

Additionally, regarding the predominance of the seated C posture in hypermobile individuals, cervical muscular characteristics such as laxity, excessive elongation, and looseness interfere with the appropriate positioning of the cervical region, which favors the anterior or lateral position of the head, which severely compromises the posture of this segment. It is important to consider that when the head is relatively anterior to the shoulders, can overload the posterior structures of the cervical spine. Ideally, from the side view, a person’s ears should align with their shoulders, placing the head 1/3 posterior to the shoulder line and 2/3 in front. In a seated posture, while using a computer or reading a book, the head is positioned forward, with 100% of the head’s weight in front of the shoulder line. This is a condition that generates overload of the posterior cervical region [50].

The present study identified that sitting in C, W, F, and S positions are characteristics that can be identified either currently or in the past in children. It was also found that the majority of children sit or have sat in W and C positions, with just under half of the sample sitting or having sat in S and F positions. Among those who have always sat with these postures, the majority are female.

The consequences of JH are reflected in adult life, as observed by deformities in the locomotor system [29,33]. Many individuals have undergone surgeries [33]. However, they are preventable and treatable, and minimize the manifestations, which can significantly alter the health of this population [7,33,47]. This starts with identifying habits, postures, and movement skills from early childhood, as presented in this study.

Given the importance of the gradual maturation of locomotor system tissues and the effects of inadequate postures in childhood on the anatomical development of the locomotor system [15,19,20,21], and the physical deformities/deficiencies observed in elderly individuals with JH [33], it is crucial for pediatricians, family doctors, physiotherapists, educators, and parents to pay attention to identifying these sitting postures. These positions, beyond causing physical health issues throughout life, also serve as a warning for identifying JH manifestations that are not benign. Special attention is needed during the growth spurt, particularly regarding inappropriate postural habits during static and dynamic activities, including sitting, lying down, and standing positions [33]. However, it is possible to promote health through actions that begin preferably in childhood [47].

The condition of children with JH, who can assume atypical positions from a seated posture as demonstrated in this study, raises questions about the implications for the development and formation of intra- and peri-articular structures due to joint instabilities, tissue fragility, and increased exposure to trauma, with potential implications in adulthood. Studies are needed to follow up on these children to analyze physical characteristics and damage in adulthood, including investigations of those who have undergone hip and/or knee prostheses. In this way of postural atypicalities inherent to JH, attention to atypical postures is necessary [31].

The positioning of the pelvis and hip is maintained by force systems acting on the pelvis, involving combined actions of the posterior trunk muscles synchronized with the anterior limb muscles and concurrent actions of the lateral pelvic muscles [51]. Hip pain is common in patients with EDS and can result from sacroiliac dysfunction, and iliotibial band subluxation over the greater trochanter, which may lead to trochanteric bursitis due to JI [30]. The fundamentals of hip mechanics justify the concern with the postures and atypical skills observed in this study.

Considering the mechanisms that define the anatomy of the locomotor system during different stages of life development [14,15,16,17,18], it is expected that these inadequate habits in children, involving unconventional sitting postures, will lead to structural changes until the end of growth, resulting in physical abnormalities. These could explain signs and symptoms in the locomotor system due to changes in the musculoskeletal architecture, influencing the phenotype.

As demonstrated in this study and also referenced by another recent study [31], the first signs of JH are present from childhood, and may persist into adulthood. Therefore, early recognition of the detrimental effects of inappropriate postures/skills in children is crucial. In this context, healthcare professionals and educators need to extend their actions to include families, aiming to raise awareness about potential physical health implications.

Poor body habits during childhood are suggestive of causing anatomical structural changes, including bone degeneration and even bone failure. Constant irritative pressures, such as those occurring in postural deformities, can lead to bone atrophy and intermittent stress from normal activities, promoting bone growth, such as walking, sitting, and standing. Conversely, appropriate weight bearing stimulates osteogenesis, depending on the piezoelectric effect [52].

The conditions related to atypical postures and body skills demonstrated in this study highlight that there are phenotypic characteristics and specificities in children that enable the identification of signs of JH and related characteristics for early intervention. Addressing inappropriate habits, postures, and body skills during the growth process is crucial to promoting physical health into adulthood. Specialized literature indicates that children exhibit signs and symptoms related to JH, which pose challenges for effective management by therapists. Physiotherapy is recognized as a central component of the multidisciplinary team in managing these children due to the implications for body mechanics [27].

The strengths of this study include the identification of phenotypic characteristics that are easily identified by health professionals, educators, and family members through the inspection of atypical body behaviors that other children may not present. According to recent literature, early recognition and proactive and appropriate management during childhood can reduce the impact of this debilitation in adulthood. It is important to mitigate the impacts related to the health conditions of hypermobile patients [47]. Thus, it is highlighted that the identification of JH characteristics in children, combined with physical therapy interventions, is relevant and feasible. Such interventions aim to maintain physical health functionality throughout the aging process of the locomotor system, thus reducing potential damage to joint mechanics and improving quality of life. It is worth mentioning that another strong point of this study is that a study published in 2005 by Lamari et al. [3] with 1120 children was the first to draw attention to the issues related to JH and the need for adjustments in the analysis of JH in children. This study was included in the 2024 meta-analysis regarding the BS parameter for children [53].

## 5. Conclusions

The ability to sit in the S, F, W, and C positions are preclinical phenotypic characteristics of JH, easily identified by pediatricians and family members to avoid possible joint deformities in the future. Most sat or sat in C and W, and just under half of them sat in S and F. BS ≥ 6 is more frequently observed in all positions. The majority of the total sample has BS > 6, with a significant influence of the female sex, and half of them have the highest BS values when compared to boys, with the difference being significant. Among those with a history of occasional joint dislocations and subluxations, half of them had the highest BS scores. It is recommended that these variables be considered in addition to the analysis of JH by BS to identify preclinical signs that may be re-educated by early physiotherapeutic assistance.

### Limitations of the Study

The Beighton Score is the most commonly used tool in clinical practice and research to identify JH [11]. The characteristics of JH are poorly understood, and even less so are its clinical implications. However, the analysis of other body regions, such as the hips, which have significant clinical relevance, is not performed, despite their frequent involvement. Thus, there are no population studies to compare with our results, except for a study on a sample that used different age groups [31].

Limitations due to retrospective and descriptive design restrict the ability to establish causal relationships or perform age-adjusted analyses. Besides, the lack of longitudinal data further limits insights into the progression of the identified preclinical signs of JH and their long-term implications. Lastly, even though the study sample included children from various regions of Brazil, the findings may not be generalizable to populations from different cultural or environmental contexts.

## Figures and Tables

**Table 1:** Descriptive statistics of BS in the total sample and according to the female and male sex.

Group	*n*	Mean ($\bar{x}$)	Standard Deviation (*s*)	*CV* (%)	Median	Range	*p*-Value Mann–Whitney U Test
Total	124	6.70	1.76	26.2	7.0	2.0–9.0	
Female	74	6.95	1.73	24.9	7.0	2.0–9.0	0.046
Male	50	6.34	1.76	27.7	6.0	2.0–9.0	

**Table 2:** Association between BS ≤ 6 and BS > 6 and total sample categories and female and male sex.

Sex	BS ≤ 6	BS > 6	Total
Female	29 (39.2%)	45 (60.8%)	74
Male	30 (60.0%)	20 (40.0%)	50
	*p*-value = 0.024 (Fisher’s test)		

**Table 3:** Percentage distribution related to ability to sit in splits (S), with the foot on the head (F), in W (W), in concave shape (C) positions according to sex.

Sitting Position (*n*)	Female	Male
S (*n* = 18)	11 (61.1%)	07 (38.9%)
W (*n* = 48)	29 (60.4%)	19 (39.6%)
F (*n* = 28)	17 (60.7%)	11 (39.3%)
C (*n* = 70)	38 (54.3%)	32 (45.7%)

## Data Availability

The original contributions presented in this study are included in the article. Further inquiries can be directed to the corresponding author.

## References

[1] Grahame R. (1999). Joint hypermobility and genetic collagen disorders: Are they related?. Arch. Dis. Child..

[2] Malfait F., Francomano C., Byers P., Belmont J., Berglund B., Black J., Bloom L., Bowen J.M., Brady A.F., Burrows N.P. (2017). The 2017 International Classification of the Ehlers-Danlos Syndromes. Am. J. Med. Genet. C Semin. Med. Genet..

[3] Lamari N.M., Chueire A.G., Cordeiro J.A. (2005). Analysis of joint mobility patterns among preschool children. São Paulo Med. J..

[4] Stern C.M., Pepin M.J., Stoler J.M., Kramer D.E., Spencer S.A., Stein C.J. (2017). Musculoskeletal Conditions in a Pediatric Population with Ehlers-Danlos Syndrome. J. Pediatr..

[5] Lamari M.M., Lamari N.M., de Medeiros M.P., Giacomini M.G., Santos A.B., de Araújo Filho G.M., Goloni-Bertollo E.M., Pavarino É.C. (2024). Generalized Joint Hypermobility: A Statistical Analysis Identifies Non-Axial Involvement in Most Cases. Children.

[6] Lamari N.M., Lamari M.M. (2016). Characterization of brazilian children with joint hypermobility. Int. J. Physiatry.

[7] Tofts L.J., Simmonds J., Schwartz S.B., Richheimer R.M., O’Connor C., Elias E., Engelbert R., Cleary K., Tinkle B.T., Kline A.D. (2023). Pediatric joint hypermobility: A diagnostic framework and narrative review. Orphanet J. Rare Dis..

[8] Kirby A., Davies R. (2007). Developmental Coordination Disorder and Joint Hypermobility Syndrome?—Overlapping disorders? Implications for research and clinical practice. Child Care Health Dev..

[9] Castori M., Tinkle B., Levy H., Grahame R., Malfait F., Hakim A. (2017). A framework for the classification of joint hypermobility and related conditions. Am. J. Med. Genet. C Semin. Med. Genet..

[10] Beighton P., Solomon I., Soskolne L. (1973). Articular mobility in an African population. Ann. Rheum. Dis..

[11] Juul-Kristensen B., Schmedling K., Rombaut L., Lund H., Engelbert R.H.H. (2017). Measurement Properties of Clinical Assessment Methods for Classifying Generalized Joint Hypermobility-A Systematic Review. Am. J. Med. Genet. C Semin. Med. Genet..

[12] Grahame R. (2013). Joint hypermobility: Emerging disease or illness behaviour?. Clin. Med..

[13] Castori M. (2021). Deconstructing and reconstructing joint hypermobility on an evo-devo perspective. Rheumatology.

[14] Purvis T. (2017). Mecânica Articular I. Traduzido por Mariane M. Franceschi Malucelli.

[15] Guedes D.P. (2011). Crescimento e desenvolvimento aplicado à Educação Física e ao esporte. Rev. Bras. Educ. Física Esp..

[16] Kłodowski A., Rantalainen T. (2015). Multibody Approach to Musculoskeletal and Joint Loading. Arch. Comput. Methods Eng..

[17] Pinheiro M.B., Avelar B.S., Teixeira-Salmela L.F. (2013). Implicações clínicas das respostas dos tecidos musculares e conjuntivos ao estresse físico. Ter. Man..

[18] Levangie P.K., Norkin C.C. (2011). Joint Structure and Function: A Comprehensive Analysis.

[19] Guedes D.P., Guedes J.E.R.P. (2000). Crescimento Composição Corporal e Desempenho Motor: De Crianças e Adolescentes.

[20] Malina R.M., Bouchard C. (2002). Atividade Física do Atleta Jovem: Do Crescimento à Maturação.

[21] Matsudo S.M., Paschoal V.C.P., Amancio O.M.S. (1997). Atividade Física e sua relação com o crescimento e a maturação biológica de crianças. Cad. Nutr. Soc. Bras. Aliment. Nutr..

[22] Galli M., Cimolin V., Vismara L., Grugni G., Camerota F., Celletti C., Albertini G., Rigoldi C., Capodaglio P. (2011). The effects of muscle hypotonia and weakness on balance: A study on Prader-Willi and Ehlers-Danlos syndrome patients. Res. Dev. Disabil..

[23] Galli M., Cimolin V., Rigoldi C., Castori M., Celletti C., Albertini G., Camerota F. (2011). Gait strategy in patients with Ehlers-Danlos syndrome hypermobility type: A kinematic and kinetic evaluation using 3D gait analysis. Res. Dev. Disabil..

[24] Rombaut L., Malfait F., De Wandele I., Cools A., Thijs Y., De Paepe A., Calders P. (2011). Medication, surgery, and physiotherapy among patients with the hypermobility type of Ehlers-Danlos syndrome. Arch. Phys. Med. Rehabil..

[25] Booshanam D.S., Cherian B., Joseph C.P., Mathew J., Thomas R. (2011). Evaluation of posture and pain in persons with benign joint hypermobility syndrome. Rheumatol. Int..

[26] Vařeková R., Vařeka I., Janura M., Svoboda Z., Elfmark M. (2011). Evaluation of Postural Asymmetry and Gross Joint Mobility in Elite Female Volleyball Athletes. J. Hum. Kinet..

[27] Scheper M.C., Juul-Kristensen B., Rombaut L., Rameckers E.A., Verbunt J., Engelbert R.H. (2016). Disability in Adolescents and Adults Diagnosed with Hypermobility-Related Disorders: A Meta-Analysis. Arch. Phys. Med. Rehabil..

[28] Chopra P., Tinkle B., Hamonet C., Brock I., Gompel A., Bulbena A., Francomano C. (2017). Pain management in the Ehlers-Danlos syndromes. Am. J. Med. Genet. C Semin. Med. Genet..

[29] Tibbo M.E., Wyles C.C., Houdek M.T., Wilke B.K. (2019). Outcomes of Primary Total Knee Arthroplasty in Patients With Ehlers-Danlos Syndromes. J. Arthroplast..

[30] Ericson W.B., Wolman R. (2017). Orthopaedic management of the Ehlers-Danlos syndromes. Am. J. Med. Genet. C Semin. Med. Genet..

[31] Lamari M.M., Lamari N.M., Araujo-Filho G.M., Medeiros M.P., Marques V.R.P., Pavarino E.C. (2022). Psychosocial and motor characteristics of patients with hypermobility. Front. Psychiatry.

[32] Sanders J.O., Qiu X., Lu X., Duren D.L., Liu R.W., Dang D., Menendez M.E., Hans S.D., Weber D.R., Cooperman D.R. (2017). The Uniform Pattern of Growth and Skeletal Maturation during the Human Adolescent Growth Spurt. Sci. Rep..

[33] Lamari N., Beighton P. (2023). Hypermobility in Medical Practice.

[34] Engelbert R.H., Juul-Kristensen B., Pacey V., de Wandele I., Smeenk S., Woinarosky N., Sabo S., Scheper M.C., Russek L., Simmonds J.V. (2017). The evidence-based rationale for physical therapy treatment of children, adolescents, and adults diagnosed with joint hypermobility syndrome/hypermobile Ehlers Danlos syndrome. Am. J. Med. Genet. C Semin. Med. Genet..

[35] Norkin C.C., White D.J. (1997). Medida do Movimento Articular: Manual de Goniometria. Artes. Medicas.

[36] Thomson A., Skneeer Pierct J. (1994). Fisioterapia de Tidy.

[37] Jekel J.F., Elmore J.G., Katz D.L. (2002). Epidemiologia, Bioestatística e Medicina Preventiva.

[38] Minitab, Inc (2010). Minitab 16.2.2 [Software].

[39] Miller S.M.C., Lamari M.M., Lamari N.M. (2015). Síndrome de Ehlers-Danlos -tipo hipermobilidade: Estratégias de inclusão. Arq. Ciênc. Saúde.

[40] Romeo D.M., Venezia I., De Biase M., Ascione F., Lala M.R., Arcangeli V., Mercuri E., Brogna C. (2022). Developmental Coordination Disorder and Joint Hypermobility in Childhood: A Narrative Review. Children.

[41] Geuze R.H. (2005). Postural Control in Children with Developmental Coordination Disorder. Neural Plast..

[42] Casanova E.L., Baeza-Velasco C., Buchanan C.B., Casanova M.F. (2020). The Relationship between Autism and Ehlers-Danlos Syndromes/Hypermobility Spectrum Disorders. J. Pers. Med..

[43] Lamari N.M., Baeza-Velasco C., Araújo Filho G.M., Lamari M.M., Medeiros M.P. (2021). Autism spectrum disorder and Ehlers-Danlos syndrome—Hypermobility type: A case report. Arch. Health Sci..

[44] Alter M.J. (2010). Ciência da Flexibilidade.

[45] Castori M., Camerota F., Celletti C., Grammatico P., Padua L. (2010). Ehlers-Danlos syndrome hypermobility type and the excess of affected females: Possible mechanisms and perspectives. Am. J. Med. Genet. Part A.

[46] Malek S., Reinhold E.J., Pearce G.S. (2021). The Beighton Score as a measure of generalised joint hypermobility. Reumatol. Int..

[47] Nicholson L.L., Simmonds J., Pacey V., De Wandele I., Rombaut L., Williams C.M., Chan C. (2022). International Perspectives on Joint Hypermobility: A Synthesis of Current Science to Guide Clinical and Research Directions. J. Clin. Rheumatol..

[48] Nicholson L.L., Chan C., Tofts L., Pacey V. (2022). Hypermobility syndromes in children and adolescents: Assessment, diagnosis and multidisciplinary management. Aust. J. Gen. Pract..

[49] Winter D.A. (2009). Biomechanics and Motor Control of Human Movement.

[50] Chiodelli L., Pacheco A.B., Missau T.S., Silva A.M.T., Corrêa E.C.R. (2015). Influence of generalized joint hypermobility on temporomandibular joint, mastication and deglutition: A cross-sectional study. Rev. CEFAC.

[51] Kapandji A.I. (2009). Fisiologia Articular. Vol. 3. Esquemas Comentados de Mecânica Humana.

[52] Whiting W.C., Zernicke R.F. (2008). Biomechanics of Musculoskeletal Injury.

[53] Williams C.M., Welch J.J., Scheper M., Tofts L., Pacey V. (2024). Variability of joint hypermobility in children: A meta-analytic approach to set cut-off scores. Eur. J. Pediatr..

